# Comparative Genome Sequence Analysis of *Choristoneura occidentalis* Freeman and *C. rosaceana* Harris (Lepidoptera: Tortricidae) Alphabaculoviruses

**DOI:** 10.1371/journal.pone.0068968

**Published:** 2013-07-05

**Authors:** David K. Thumbi, Catherine Béliveau, Michel Cusson, Renée Lapointe, Christopher J. Lucarotti

**Affiliations:** 1 Natural Resources Canada, Canadian Forest Service – Atlantic Forestry Centre, Fredericton, New Brunswick, Canada; 2 Natural Resources Canada, Canadian Forest Service – Laurentian Forestry Centre, Québec, Quebec, Canada; 3 Sylvar Technologies Inc., Fredericton, New Brunswick, Canada; Northeast Agricultural University, China

## Abstract

The complete genome sequences of *Choristoneura occidentalis* and *C. rosaceana* nucleopolyhedroviruses (ChocNPV and ChroNPV, respectively) (*Baculoviridae*: *Alphabaculovirus*) were determined and compared with each other and with those of other baculoviruses, including the genome of the closely related *C. fumiferana* NPV (CfMNPV). The ChocNPV genome was 128,446 bp in length (1147 bp smaller than that of CfMNPV), had a G+C content of 50.1%, and contained 148 open reading frames (ORFs). In comparison, the ChroNPV genome was 129,052 bp in length, had a G+C content of 48.6% and contained 149 ORFs. ChocNPV and ChroNPV shared 144 ORFs in common, and had a 77% sequence identity with each other and 96.5% and 77.8% sequence identity, respectively, with CfMNPV. Five homologous regions (*hrs*), with sequence similarities to those of CfMNPV, were identified in ChocNPV, whereas the ChroNPV genome contained three *hrs* featuring up to 14 repeats. Both genomes encoded three inhibitors of apoptosis (IAP-1, IAP-2, and IAP-3), as reported for CfMNPV, and the ChocNPV IAP-3 gene represented the most divergent functional region of this genome relative to CfMNPV. Two ORFs were unique to ChocNPV, and four were unique to ChroNPV. ChroNPV ORF chronpv38 is a eukaryotic initiation factor 5 (*eIF-5*) homolog that has also been identified in the *C. occidentalis* granulovirus (ChocGV) and is believed to be the product of horizontal gene transfer from the host. Based on levels of sequence identity and phylogenetic analysis, both ChocNPV and ChroNPV fall within group I alphabaculoviruses, where ChocNPV appears to be more closely related to CfMNPV than does ChroNPV. Our analyses suggest that it may be appropriate to consider ChocNPV and CfMNPV as variants of the same virus species.

## Introduction

Baculoviruses are pathogens specific to insects in the orders Lepidoptera, Diptera, and Hymenoptera [1]. They are distinguished by their rod-shaped nucleocapsids, which are either singly or multiply enveloped, and then embedded in proteinaceous capsules known as occlusion bodies (OBs). OBs are produced during the late phase of the viral replication cycle and are composed of either polyhedrin or granulin protein, hence the respective names nucleopolyhedrovirus (NPV) and granulovirus (GV). Collectively, baculoviruses are classified in the family *Baculoviridae*, which consists of four genera. Lepidopteran-specific NPVs and GVs are grouped into the genera *Alphabaculovirus* and *Betabaculovirus*, respectively [1]. In these genera, the replication cycle is characterized by production of two morphologically distinct, but genotypically identical, virion phenotypes. The budded virion (BV) phenotype is produced during the early phase of viral replication and is involved in systemic infection of host tissues. The occlusion-derived virion (ODV) phenotype is produced during the late phase of viral replication and is involved in the horizontal transmission of the virus within host populations. Hymenopteran and dipteran NPVs are grouped into the genera *Gammabaculovirus* and *Deltabaculovirus*, respectively [1]. Both genera are considered to be more ancient than the lepidopteran baculoviruses, and gammabaculoviruses have the smallest baculovirus genomes sequenced to date and do not appear to produce a BV phenotype [2], [3]. To date, only the *Culex nigripalpus Deltabaculovirus* (CuniNPV) has been sequenced [4] and it was shown to encode an OB protein that is structurally distinct from polyhedrin and granulin proteins [5].

As a family, the *Baculoviridae* display several common genomic features including: i) large, circular, covalently closed, double-stranded DNA, ii) bidirectional and random distribution of open reading frames (ORFs) on both DNA strands, iii) 37 core genes common to all species [6], iv) promoters that regulate a temporal cascade of gene expression, and v) host cell nucleus-centered replication of genomes. An increasing number of baculovirus genomes are being sequenced, and those sequenced to date range in size from 81.7 kbp, for *Neodiprion lecontei* NPV (NeleNPV) [2], to 178.7 kbp for *Xestia c-nigrum* GV (XcGV) [7]. Baculoviruses have been widely used as environmentally benign biological control agents for insect pests [8], [9] and in biomedical platforms [10], [11].

Members of the genus *Choristoneura* (Lepidoptera: Tortricidae) are holarctic in distribution [12], and many are important defoliators of conifers. In North America, the spruce budworm (SBW), *C. fumiferana*, is the major defoliating insect pest of coniferous forests, especially in eastern Canada [13], where it exhibits prolonged, cyclical, population outbreaks [14], [15]. The western SBW (wSBW), *C. occidentalis*, occurs west of the Rocky Mountains from central British Columbia to New Mexico [16], [17] and is a major defoliator of Douglas fir (*Pseudotsuga menziesii*) and other conifers in western North America [18]. The specific epithet for *C. occidentalis* has recently been challenged and *C. freemani* suggested as a replacement [19]. Here, we use *C. occidentalis* sensu Freeman [20] as this appears to be the current practice [17], [21]. The obliquebanded leafroller (OBL), *C. rosaceana*, is a trans-continental native of North America and a major economic pest of deciduous fruit trees in Canada and the United States; it has demonstrated resistance to various broad-spectrum insecticides [22]. Although *C. fumiferana* and *C. occidentalis* belong to a group of closely related conifer-feeding budworms known as the *C. fumiferana* species complex, *C. rosaceana* is clearly an outgroup species [17].

The genomes of two NPVs infecting *C. fumiferana*, CfMNPV [23] and CfDEFNPV [24] have been sequenced. Although CfDEFNPV was considered “defective” due to its inability to infect SBW by the *per os* route, it has been postulated to synergize CfMNPV infectivity through an unknown mechanism [24]. Field surveys of SBW populations in New Brunswick, Canada [25] revealed that these populations had low prevalence of CfMNPV and ChfuGV (2% and 15%, respectively) [26]. Although few patent baculovirus infections were identified in these SBW, it has recently been reported that field-collected and laboratory-reared SBW had high prevalence of single and mixed covert infections of CfMNPV, CfDEFNPV and a GV [27]. In contrast to the low prevalence of baculoviruses in SBW populations in New Brunswick [26], diagnosis of field-collected wSBW larvae from British Columbia showed high levels of mortality (up to 70%) due to ChocNPV and other entomopathogens [18]. To date, only the wSBW betabaculovirus (ChocGV) genome has been sequenced [28]. However, a previous study reported that three *Choristoneura* GVs, isolated from *C. fumiferana*, *C. occidentalis*, and *C. retiniana*, had only minor differences in their restriction endonuclease–gel electrophoresis (REN) patterns, suggesting they were potential variants of the same GV [29]. The studies of New Brunswick SBW populations [25], [26] also identified an alphabaculovirus infection in OBL larvae that was distinct from CfMNPV [30]. The balsam fir (*Abies balsamea*)–SBW food web supports myriad parasitoids and pathogens where species of viruses are, at least numerically, a small component [25]. Genomic studies of *Choristoneura* baculoviruses may provide important additional information on the differential prevalence and roles of baculovirus infections in different *Choristoneura* species, and the evolutionary relationships between these viruses and the *Choristoneura* species complex [17], [21]. Here, we report on genome sequence analyses of two alphabaculoviruses, ChocNPV and ChroNPV, and their comparison with CfMNPV and other baculovirus genomes.

## Methods

### Virus Amplification and DNA Extraction

The wild-type ChocNPV was isolated from wSBW larvae collected from the field in British Columbia, Canada in 2007 [18] (ChocNPV_BC1, GenBank accession number KC961303). ChroNPV was isolated from OBL larvae collected near Saint-Quentin, New Brunswick, Canada in 1992 [26], [30] (ChroNPV_NB1, GenBank accession number KC961304). To obtain sufficient viral stocks for genomic work, both wild-type ChocNPV and ChroNPV were separately amplified in their respective hosts as previously described [30]. Viral OBs were purified from larval cadavers, and DNA extracted from ODVs as previously described [31], [32]. Purity of viral DNA was ascertained using a NanoDrop spectrophotometer (Thermo Fisher Scientific, Wilmington, Delaware, USA) and REN analysis.

### Genome Sequencing and Analysis

Shotgun sequencing of ChocNPV and ChroNPV genomes was done on a Roche 454 GS-FLX sequencer at IBIS (Institut de biologie intégrative et des systèmes, Université Laval, Québec, Canada). Contig assembly was carried out using SeqManPro (Lasergene DNAStar software package), and the complete sequence was obtained by ordering contigs using BioEdit [33]. The remaining gaps were filled in by PCR amplification and Sanger sequencing of the purified amplicons. Putative ORFs were identified with the sorted six-frame translation tool in BioEdit, with ORF size threshold of 50 amino acids [33], [34]. Genome annotation was done using Artemis software [35]. Putative baculoviral homologs of ChocNPV and ChroNPV ORFs were identified by searching the NCBI non-redundant protein database using the blastp and psi-blast algorithms [36], [37], [38]. Homologous regions (*hrs*) were identified and analyzed based on the consensus palindromic repeats [39] common in most baculovirus genomes. Global alignment of ChocNPV and CfMNPV sequences was done using EMBOSS Stretcher analysis (http://www.ebi.ac.uk/Tools/psa) and dot matrix analysis. To identify the most divergent sections of the alignments, both genomes were compared using the wgVista tool (http://genome.lbl.gov/vista/index.shtml). A baculovirus phylogenetic tree was generated using concatenated baculovirus LEF-8 and PIF-2 amino acid sequences [40] that were available at the time of analysis. A phylogenetic tree for a eukaryotic initiation factor 5 (EIF-5) was generated by aligning the amino acid sequence of ChroNPV ORF chronpv38 with 18 homologs obtained from the NCBI database including those of ChocGV, *Apis mellifera, Bombus impatiens, Camponotus floridanus, Acromymex echinatior, Solenopsis invicta, Nasonia vitripennis, Acyrthosiphon pisum, Tribolium castaneum, Danus plexippus, Bombyx mori, Aedes aegypti, Anopheles gambiae, Drosophila melanogaster, Drosophila persimilis, Homo sapiens, Bos Taurus,* and *Schizosaccharomyces pombe.* The trees were inferred using the MEGA5 software [41] and the UPGMA method [42], with a bootstrap analysis of 1000 pseudo-replicates [43].

## Results and Discussion

### Nucleotide Sequence Analysis

The ChocNPV genome (200 times 454 sequencing coverage) was calculated to be 128,446 bp in length, 1147 bp smaller than the CfMNPV genome, with a G+C content of 50.1%, which is similar to that of CfMNPV [23]. In comparison, the ChroNPV genome (140 times 454 sequencing coverage) was found to be 129,052 bp in length, 606 bp larger than ChocNPV but 541 bp smaller than CfMNPV, with a G+C content of 48.6%. Based on the convention for identifying putative baculovirus ORFs [36], a total of 148 and 149 ORFs showing minimal overlap and encoding putative proteins of 50 amino acids or more were identified in ChocNPV and ChroNPV genomes, respectively. There were, however, a few exceptions with overlap. For example, ChocNPV showed large overlaps of up to 416 bp in two contiguous ORFs, chocnpv107 and chocnpv108. This observation was consistent with that previously reported for the CfMNPV genome [23]. In the ChroNPV genome, however, the largest overlap spanned only 152 bp between ORFs chronpv95 and chronpv96, which encode the *lef10* and *vp1050* genes, respectively. Similar size overlaps occur in the CfMNPV and ChocNPV genomes, but between different genes. Overall, the coding sequences in ChocNPV accounted for 92.9% of the entire genome, similar to what was reported for the *Epiphyas postvittana* MNPV (EppoMNPV) genome [44]. In comparison, the ChroNPV coding sequences accounted for 94.1% of the entire genome.

**Figure 1:**
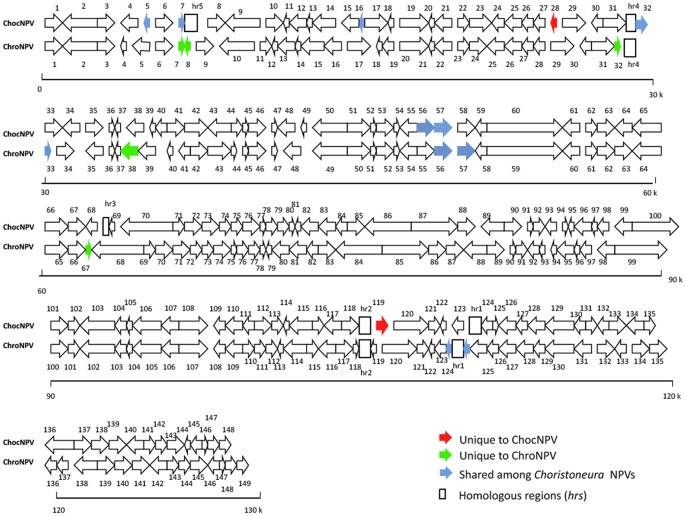
Linear representation of ChocNPV and ChroNPV genomes. The arrows depict the respective 148 and 149 putative ORFs and their relative positions in ChroNPV and ChocNPV genomes. ORFs unique to ChocNPV and ChroNPV genomes are indicated in red and green arrows, respectively. Blue arrows represent shared ORFs, whereas open boxes represent the relative positions of homologous regions (*hrs*) in the two genomes.

**Figure 2:**
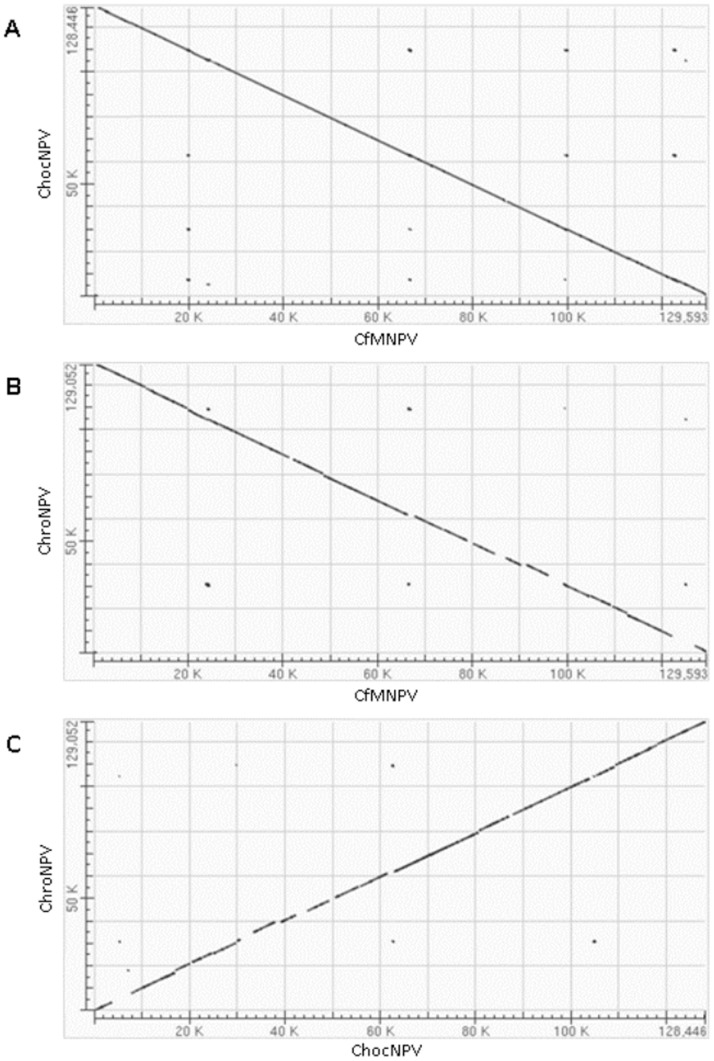
Dot matrix analysis of three *Choristoneura* NPV genomes. The plots were generated using blastN global genome alignment and compare; A) ChocNPV and CfMNPV, B) ChroNPV and CfMNPV, and C) ChroNPV and ChocNPV genomes. All genomes show a high degree of co-linearity.

In both ChocNPV and ChroNPV genomes, putative ORFs were sequentially numbered starting from the first methionine of the *polyhedrin* gene with a forward orientation. A different convention was used for six other sequenced baculovirus genomes (CfMNPV [23], CfDEFNPV [24], EppoMNPV [44], OpMNPV [45], AgMNPV [46], and AnpeNPV [47]), for which the numbering was done in the opposite direction. The forward:reverse ratio for ChocNPV ORFs was close to 1∶1, with 72 in forward and 76 in reverse orientations (Figure 1 and Table S1). Similarly, ChroNPV had 75 ORFs on the positive strand and 74 on the negative strand (Figure 1 and Table S2). This distribution is consistent with that reported for most baculovirus genomes, except for the hymenopteran gammabaculoviruses, which have a forward:reverse ORF ratio closer to 6∶4 [2], [48], [49]. As observed for other baculovirus genomes, no physical clustering of ChocNPV and ChroNPV ORFs was found as a function of their temporal expression or putative roles. Global alignment and dot matrix analysis showed co-linearity between CfMNPV and ChocNPV and an overall nucleotide identity of 96.5%. Nucleotide identity values were lower for the CfMNPV-ChroNPV (77.8%) and ChocNPV-ChroNPV (77%) comparisons (Figure 2).

### Homologous Regions (*hrs*)

Most baculovirus genomes sequenced to date contain from 1 to 16 *hrs* [50]. These DNA elements are interspersed throughout the genome and have been implicated as putative origins of DNA replication (*oris)*, as enhancers of gene transcription, and in homologous recombination [51]. Although their architecture varies among baculovirus species, most *hrs* have palindromic sequence motifs located at the core of several repeat units [51]. In addition to *hrs*, some baculovirus genomes harbor repetitive sequences designated as *non-hr*. Previous studies implicated *non-hr* as putative *oris*, particularly due to their prevalence in defective interfering particles (DIs) following continued serial passage of baculoviruses and their differential *in vivo* activities [52], [53]. Neither ChocNPV nor ChroNPV were found to contain *non-hr* elements, thus, these two viruses may use *hrs* or some other unknown sequence as origins of replication. The *hrs* in both genomes, however, differed in terms of their number, number of repeats, and distribution relative to other baculovirus genomes (Table 2). The ChocNPV genome contained five *hrs* with an average length of 365 bp, representing 1.4% of the entire genome sequence. These *hrs* were located in the same genomic loci as their CfMNPV counterparts. The ChroNPV genome, on the other hand, contained three *hrs* featuring 3–14 repeats representing 1.6% of the entire genome sequence. As previously noted, gene rearrangements and/or acquisitions are common occurrences around *hrs* in some baculoviruses [23]. This possibility was also noted in both ChocNPV and ChroNPV genomes. For example, two ChroNPV ORFs, chronpv124 and chronpv125, with respective amino acid identities of 33% and 39% relative to CfMNPV ORF116, flanked and overlapped *hr1* (Table 2). Also, ChroNPV ORF chronpv118 overlapped *hr2* and exhibited 25% amino acid identity with CfMNPV ORF Cf116. In ChocNPV, chocnpv7 overlapped *hr5* and chocnpv32 overlapped *hr4* with respective amino acid identities to CfMNPV ORF Cf116 of 29% and 89%. Based on these observations, it appears that ChocNPV and ChroNPV may have acquired these ORFs from a shared host. In addition, some unique ORFs, such as chronpv32 and chocnpv119, were adjacent to and overlapped *hrs* (Table 2), supporting the possibility of gene transfer among viruses or between viruses and hosts via homologous recombination. The prevalence of numerous pathogens [25] as well as mixed covert and overt baculoviral infections in field populations of SBW [27] could have facilitated these gene transfers.

**Table 2:** Features of ChocNPV and ChroNPV *hrs.*

Virus	Name	Sequence position (bp)	Length	No. of repeats	Comments
ChocNPV	hr1	109054–109460	406	6	no overlap
	hr2	104768–105142	374	6	overlap with chocnpv119
	hr3	62501–62761	260	4	overlap with choc69
	hr4	29640–29981	341	5	overlap with chocnpv32
	hr5	7254–7701	447	5	overlap with chocnpv7
ChroNPV	hr1	108737–109602	865	13	overlap with chronpv124 and chronpv125
	hr2	104304–104515	211	3	overlap with chronpv118
	hr4	30077–31016	939	14	overlap with chronpv32 and chronpv33

### Gene Content and Homology

**Figure 3:**
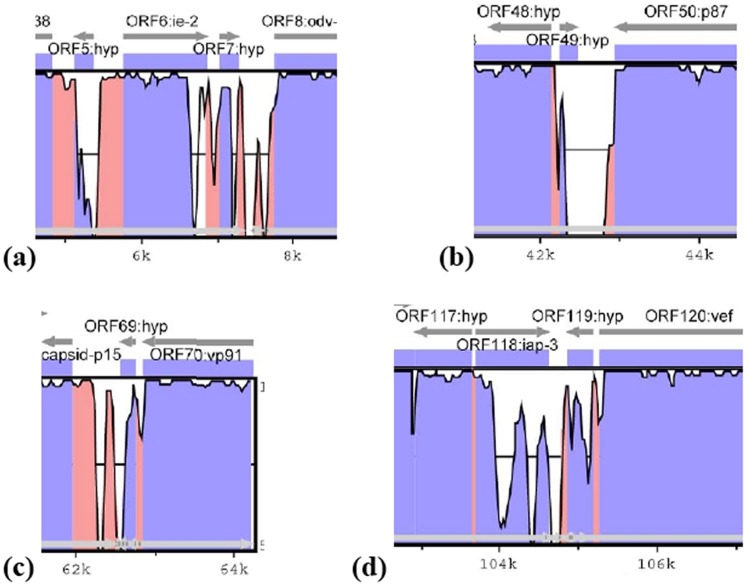
Vista analysis. Graphical representation of the most divergent regions of the ChocNPV and CfMNPV genomes. The plots were generated using wgVista tool. Blue and red colors represent coding and non-coding sequences, respectively. Peaks and valleys represent percent conservation between aligned sequences at a given coordinate. The top and bottom lines represent 100% and 50% identity, respectively. Above the top line are ORF numbers, names, and orientation. Overall, five most divergent regions were identified, with section (a), containing two regions.

Both ChocNPV and ChroNPV genomes were directly compared with five other alphabaculoviruses, namely CfMNPV, CfDEFNPV, OpMNPV, AcMNPV, and HycuNPV. Apart from AcMNPV, which is generally used as the baculovirus reference genome, the other four alphabaculoviruses were selected based on their evolutionary relatedness and similar ecological distribution to ChocNPV and ChroNPV. ChocNPV and ChroNPV genome features and similarity data (percent amino acid identity) are provided in Tables S1 and S2. Overall mean percent amino acid identity between ChocNPV or ChroNPV ORFs and baculoviral orthologues was >70%, except for AcMNPV, which displayed <58% average amino acid identity relative to ChocNPV and ChroNPV ORFs (Table 1). Based on homology searches, ChocNPV appears to be most closely related to CfMNPV, with a mean amino acid sequence identity of 97.3% compared with a mean sequence identity of 82.1% between ChroNPV and CfMNPV. ChocNPV and ChroNPV genomes shared 144 ORFs, and after accounting for differences in ORF numbering schemes, most of the ORFs identified in ChocNPV and ChroNPV were also shared with CfMNPV. Based on VISTA curve analysis, however, five regions in the alignment of ChocNPV and ChroNPV genomes were identified as being divergent (Figure 3). Regions (i) and (ii) include both ChocNPV ORFs chocnpv5 and chocnpv7 (Figure 3a), which encode hypothetical proteins displaying low amino acid identity (39% and 55%) relative to their CfMNPV orthologs, Cf143 and Cf116, respectively. In addition, homologs of these ORFs were not found in all other baculovirus genomes examined, including ChroNPV. Region (iii) begins with ORF chocnpv49 but comprises mostly non-coding sequences (Figure 3b) not present in CfMNPV or ChroNPV genomes. However, when other reading frames are considered, this region as a whole shows similarity to *he65* homologs found in a few alphabaculoviruses, including CfDEFNPV (Cfdef98), AcMNPV (ac105), and AnpeNPV (Anpe97). He65 contains an adenylation DNA ligase domain that catalyzes ligation of nicked DNA during DNA replication, repair, and recombination. Interestingly, this domain is not conserved in ChocNPV region (iii), implying a possible loss of function during evolution. Region (iv) (Figure 3c) is also primarily a non-coding sequence but includes chocnpv69, which is a hypothetical protein absent in the ChroNPV and CfMNPV genomes. Like that of chocnpv7, the chocnpv69 product exhibited weak amino acid identity (48%) relative to CfMNPV Cf116 and includes a portion of *hr3*. Finally, region (v) (Figure 3d) corresponds to chocnpv118, an inhibitor of apoptosis 3 (IAP-3) that is an ortholog of CfMNPV Cf30 and appears to be the most divergent functional region relative to CfMNPV, with only 70% amino acid identity between the two orthologs. A hypothetical protein not found in the CfMNPV genome, chocnpv118 is adjacent to *hr2*, which overlaps chocnpv119 (Table 2). As *hrs* have been implicated in homologous recombination, it is possible that the observed divergence in region (v) has occurred as a result of loss or acquisition of new genes during virus–host interactions.

**Table 1:** Characteristics of ChocNPV and ChroNPV genomes.

			Baculoviruses*					
Features	Choc	Chro	Cf	CfDEF	Op	Hycu	Ac	
Genome size (bp)	128,446	129,052	129,593	131,160	131,990	132,959	133,894	
GC content (%)	50.1	48.6	50.1	50	55	45	40	
No. of ORFs	148	149	146	149	152	148	155	
No. of *hrs*	5	3	5	13	5	6	9	
No. of *bros*	1	2	1	2	3	5	1	
Mean % aa ID with Choc	–	77	97.3	70.1	76.7	75.4	56.2	
Mean % aa ID with Chro	77	–	82.1	71.1	77.4	74.6	57.4	
No. of homologs in Choc	–	144	144	134	133	128	131	
No. of homologs in Chro	144	–	135	128	133	130	128	
ORFs unique to Choc	2		–	–	–	–	–	
ORFs unique to Chro		4	–	–	–	–	–	

* ChocNPV and ChroNPV homologs were compared with five alphabaculoviruses (CfMNPV, CfDEFMNPV, OpMNPV, HycuNPV, and AcMNPV). Amino acid identities were based on BLASTP homology search. Both ChocNPV and ChroNPV shared more ORFs with CfMNPV than with the other viruses. ChocNPV had a mean amino acid identity of 97.3% and ChroNPV 82.1% with CfMNPV.

In addition to the above differences between CfMNPV and ChocNPV homologs, some mutations and insertions/deletions (INDELS) were noted. For example, there was an insert of 66 nucleotides in chocnpv4, which encodes *pe38*– involved in viral transcription transactivition, DNA replication, BV production and oral infectivity [54], [55]. In addition, chocnpv8 (*odv-e56* or *per os* infectivity factor 5 (*pif-5*)) contained two gaps of 36 and 12 nucleotides relative to the CfMNPV homolog Cf141. Similarly, relative to CfMNPV Cf83, a hypothetical protein of unknown function, the chocnpv64 product, had two deletions of 16 and 24 amino acids.

### Genes Involved in *per os* Infection, Genome Biosynthesis, and Virus Morphogenesis

As in other systems, baculovirus genes are categorized based on their functional roles in host pathogenesis. Both ChocNPV and ChroNPV genomes contained the 37 core genes shared among all baculoviruses sequenced to date [6]. This core set of conserved genes constitutes a repertoire of factors involved in initiating infections, transcription, replication, and production of mature progeny virions. The primary mode of infection is mediated by *per os* infectivity factors (PIFs), which are components of ODVs. A number of PIF genes, *pif-0/p74*, *pif-1*, *pif-2*, *pif-3*, *pif-4/19K* (*odv-e28*), *pif-5* (*odv-e56*), ac68 [56], and ac108 homolog sf58 [57], have been reported in baculoviruses, and both ChocNPV and ChroNPV PIFs exhibited high sequence identity with other baculovirus PIF orthologs (Tables S1 and S2). BVs of alphabaculoviruses contain homologs of either GP64 or F proteins that are essential in establishing systemic infections in their hosts and may act as host range factors [58], [59]. On the basis of these membrane fusion proteins, alphabaculoviruses are divided into group I NPVs, which have GP64, and group II NPVs, which have F proteins. Both ChocNPV and ChroNPV encode GP64 homologs, placing them in group I along with CfMNPV [23] and CfDEFNPV [24].

Studies using both transient expression assays and gene knock-out approaches have elucidated the functional roles of baculovirus genes involved in transcription and DNA replication [54], [60]. Both ChocNPV and ChroNPV shared genes involved in these molecular functions that have been found in other alphabaculoviruses (Tables 3, S1 and S2). Although non-essential for DNA replication, homologs of genes involved in nucleotide metabolism and DNA repair, including the two ribonuclease reductase genes (*rr1* and *rr2*), *dUTPase*, and *DNA ligase*, were absent in both ChocNPV and ChroNPV genomes. This was not unexpected, as neither of the previously sequenced *Choristoneura* NPVs contain homologs of these genes [23], [24]. Furthermore, association of these genes in some baculovirus genomes has been shown to be phylogenetically and functionally linked [61]. In addition, homologs of helicase 2 (*hel-2*) are missing in all *Choristoneura* NPVs, although one was identified in ChocGV, along with a *dna ligase* [28]. Herniou et al. [61] pointed out that, with the exception of *Spodoptera litura* NPV (SpltNPV), *hel-2* only occurs in baculovirus genomes featuring a *dna ligase* and that both genes could be involved in DNA recombination or repair. Thus, it appears that *Choristoneura* NPVs either possess an as yet uncharacterized DNA repair system or may have lost these genes due to their non-essential roles during viral replication in *Choristoneura* insect hosts. This notion may be reinforced by the absence of an adenylation DNA ligase domain in ChocNPV conserved region (iii) (Figure 3).

**Table 3:** ChocNPV and ChroNPV genes present in other baculovirus genomes.

Category	ChocNPV & ChroNPV genes in other NPVs	Genes absent in *Choristoneura* NPVs			
**Transcription**	***lef-4*** *, * ***lef-5*** *, lef-6, lef-7, * ***lef-8*** *, * ***lef-9*** *,*				
	*lef-11, lef-12, * ***p47*** *, pe38, * ***vlf-1***				
**Replication** 1	***lef-1*** *, * ***lef-2*** *, lef-3, * ***dnapol,*** ** ***hel*** *, ie-0, ie-1, ie-2,*		*dUTPase, rr1, rr2,*		
	***38k*** *, pcna, me53, gta, v-trex, dbp, pp31/39k*		*dna ligase*		
**Structural**	*polh, cap1629, * ***odv-e27*** *, * ***odv-e18*** *, * ***p49*** *,*				
	*gp64, pep, gp16, p24, * ***odv-ec43*** *, * ***p40*** *, * ***p6.9*** *,*				
	***p33*** *, * ***vp39*** *, p15, * ***vp91*** *, * ***gp41*** *, * ***vp1054*** *, odv-e66*				
	*odv-e26, * ***ac81,*** ** ***desmoplakin, pif-0 (P74),***				
	***pif-1*** *, * ***pif-2*** *, * ***pif-3, pif-4 (odv-e28)*** *,*				
	***pif-5 (odv-e56),*** ** ***ac53*** *, * ***ac68*** *, * ***ac78*** *, * ***odv-e25*** *, fp, gp64*				
**Auxiliary** 2	*ptp-1, ptp-2, iap-1, iap-2, iap-3, bro-a, bro, egt,*		pnk/pnl (ac86)		
	*ctl-1, ctl-2, lef-10, * ***alk-exo,*** * v-cath, v-chi, p10, arif-1,*				
	*sod, fgf, v-ubi, pkip, * ***p18*** *, p26a, p26b, * ***p48*** *, p87, vef, pk-1*				
	*copia-like (ac23), tlp, met, slp, ChaB, etm, nmap, p12*				
	*cg30, elf-5*				

Genes are categorized based on their functions during virus replication. The 37 baculovirus core genes [6] are shown in bold.

1 The replication gene *v-trex* is absent in ChroNPV genome. Also missing in both ChocNPV and ChroNPV are genes involved in DNA repair system and nucleotide metabolism.

2 Highlighted in grey are auxiliary genes *ctl-2* and *elf-5* present in ChroNPV genome, but not in other *Choristoneura* NPVs.

In addition to PIFs and GP64 envelop fusion proteins, homologs of structural genes conserved in most baculoviruses [6], [61] were present in both ChocNPV and ChroNPV genomes (Table 3) and exhibited high sequence identity values with the other baculoviruses referenced (Tables S1 and S2).

### Auxiliary Genes

Although they confer selective advantage to viruses, auxiliary genes are non-essential in viral gene expression, DNA replication, and progeny virion formation [40], [59], [62]. In addition to the alkaline exonuclease gene (*alk-exo*), which is conserved in all baculoviruses, both ChocNPV and ChroNPV genomes contained homologs of auxiliary genes that have been identified in many baculovirus genomes including that of CfMNPV [23]. As is the case with other ORFs, ChocNPV auxiliary genes exhibited higher sequence identity (98.8%) with CfMNPV homologs than with their ChroNPV counterparts (88.3%). Unlike other *Choristoneura* NPVs, the ChroNPV genome contained an extra copy of a gene encoding a conotoxin-like protein (*ctl-2*), which showed high sequence identity to homologs in OpMNPV (85%) and HycuNPV (79%). Although homologs of *ctl* are implicated in calcium ion inhibition, their *in vivo* role during baculovirus replication is unclear [63]. Shared among all *Choristoneura* NPVs are homologs of protein-tyrosine phosphatase genes (*ptp-1* and *ptp-2*) and ecdysteroid UDP glucosyltransferase (*egt*). Together, these genes have been linked to enhanced locomotory activity and climbing behavior (tree top disease) in virus-infected larvae [64], [65].

### Inhibitors of Apoptosis

Apoptosis (programmed cell death) is a highly regulated biological process essential for developmental and immune responses in multicellular organisms [66]. Holometabolous insects have evolved this conserved mechanism to aid metamorphosis and defend against baculovirus infections [67]. To counteract this apoptotic host immune response, baculoviruses encode inhibitor of apoptosis (*iap*) genes and/or homologs of caspase inhibitors such as P35 and P49 [68], [69]. In addition, baculovirus IAPs have been implicated as host range determinants [70]. There are five baculovirus IAPs (IAP 1–5) grouped according to their sequence similarity [71]. Features unique to IAPs are RING-finger motifs at the carboxyl-terminus and baculovirus IAP-repeat(s) (BIRs) at the N-terminus that are involved in binding apoptosis-inducing factors through protein–protein interactions [72], [73]. As was shown for CfMNPV [23], ChocNPV and ChroNPV genomes contain three *iap*s (*iap-1*, *iap-2*, and *iap-3*). The IAPs of both ChocNPV and ChroNPV exhibited high sequence identity with their orthologs in related alphabaculoviruses, including OpMNPV, HycuNPV, and CfMNPV. However, the main difference between ChocNPV and CfMNPV was found within the *iap-3* sequence. Consistent with the previous examination of the CfMNPV genome [23], neither the ChocNPV nor the ChroNPV genome contained an ortholog of *iap-4*, found in EppoMNPV [44] and OpMNPV [45]. As for most baculoviruses, ChocNPV and ChroNPV genomes do not contain homologs of *iap-5*, which has only been identified in a few betabaculoviruses, including ChocGV [28] and those of *Pieris rapae* (PiraGV) [74] and *Adoxophyes orana* (AdorGV) [75].

### Unique ORFs

ORFs with no identifiable baculovirus homologs were found in both ChocNPV and ChroNPV genomes. Two ORFs, chocnpv28 and chocnpv119, were unique to ChocNPV, and four, chronpv7, chronpv8, chronpv32, and chronpv67, were unique to ChroNPV (Figure 1). In comparison, seven ORFs (Cf89, Cf90, Cf116, Cf120, Cf121, Cf133, and Cf143) had previously been identified as being unique to the CfMNPV genome [23]. Five of these, Cf116, Cf120, Cf121, Cf133, and Cf143, are no longer unique as they have clear homologs in ChocNPV and/or ChroNPV (Tables S1 and S2), where they are located in similar genomic positions. For example, chocnpv32 and chronpv33 are homologs of each other and Cf116 and overlap their respective *hr4*s. As all homologs of Cf116 (chocnpv7, chocnpv69, chronpv118, chronpv124 and chronpv125; Figure 1, Tables 2, S1 and S2) are linked or close to *hrs*, it is likely that Cf166 and its homologs are somehow associated with *hr* sequences.

A homolog of ChocNPV/ChroNPV ORF 122 could not be found in CfMNPV, but one was present in EppoMNPV (Eppo28), which encodes a hypothetical protein [44] showing 53% and 60% sequence identity to the proteins encoded by chocnpv122 and chronpv122, respectively. *Choristoneura* NPVs and EppoMNPV are phylogenetically related within group I alphabaculoviruses, with CfDEFNPV being most closely related to EppoMNPV. Based on previous reports on co-evolution of baculoviruses with their host [61], it is possible that the progenitor of chocnpv122 and chronpv 122 was acquired by an ancestral *Choristoneura* NPV but was then lost in CfMNPV. Another difference between ChroNPV and other *Choristoneura* NPVs is the lack of a *v-trex* gene, which is found in CfMNPV (Cf114) [23], CfDEFNPV (Cfdef119) [24], and ChocNPV (chocnpv34). Homologs of *v-trex* possess three conserved domains, EXOI, EXOII, and EXOIII, involved in 3′–5′ exonuclease activity in prokaryotic and eukaryotic DNA replication [76]. In CfMNPV and a few other alphabaculoviruses (e.g., AgMNPV), *v-trex* genes were implicated in proof-reading and DNA repair mechanisms during viral DNA replication [77], [78]. It is unclear why the ChroNPV genome lacks this gene.

### Eukaryotic Translation Initiation Factor 5

**Figure 4:**
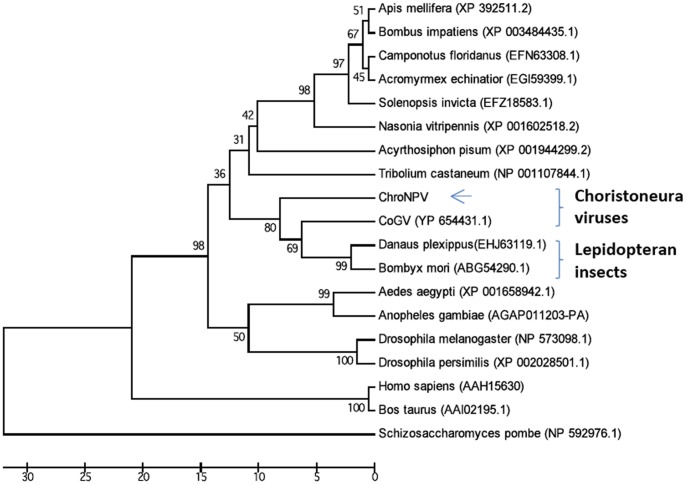
Phylogenetic tree for eukaryotic initiation factor 5 (eIF-5). Homologues of ChroNPV eIF-5 were obtained from NCBI database using BLASTP. The tree was generated based on concatenated amino acid sequences of ChroNPV eIF-5 and of other eukaryotic organisms available in the database. GenBank accession number for some analyzed taxa is shown beside those taxa. The analysis was conducted in MEGA 5 [41] and inferred using the UPGMA method [42]. The bootstrap test values (1000 pseudo-replicates) are shown next to the branches [43].

Another difference between ChroNPV and the other *Choristoneura* NPVs is the unique presence of a eukaryotic translation initiation factor 5 (eIF-5, encoded by chronpv38) displaying 72% sequence identity with ChocGV ORF 10 [28]. Homologs of eIF-5 have also been reported in various insect species, where they exhibit sequence conservation. eIF-5 acts as a translation initiation factor involved in regulation of protein synthesis. In addition, eIF-5 has been implicated as a regulator of developmental processes such as metamorphosis in holometabolous insects [79]. Expression of *Helicoverpa armigera eIF-5* (Ha-*elF5C*) in the head, thorax, integument, midgut, and fat body has been shown to increase during metamorphosis [79]. Although the molecular function of ChroNPV *eIF-5* is unclear, this gene may encode a protein involved in the interference of host physiological or immune responses. Similar translational regulatory factors have been shown to act as inhibitors of host translational machinery to the benefit of viral translation [80]. Neither ChocNPV nor CfMNPV appear to contain an *eIF-5* ortholog, but its presence in the ChroNPV genome and its phylogenetic relationship to insect *eIF-5* homologs (Figure 4) suggest that it is the product of horizontal gene transfer through host–pathogen interactions.

### Phylogenetic Analysis

**Figure 5:**
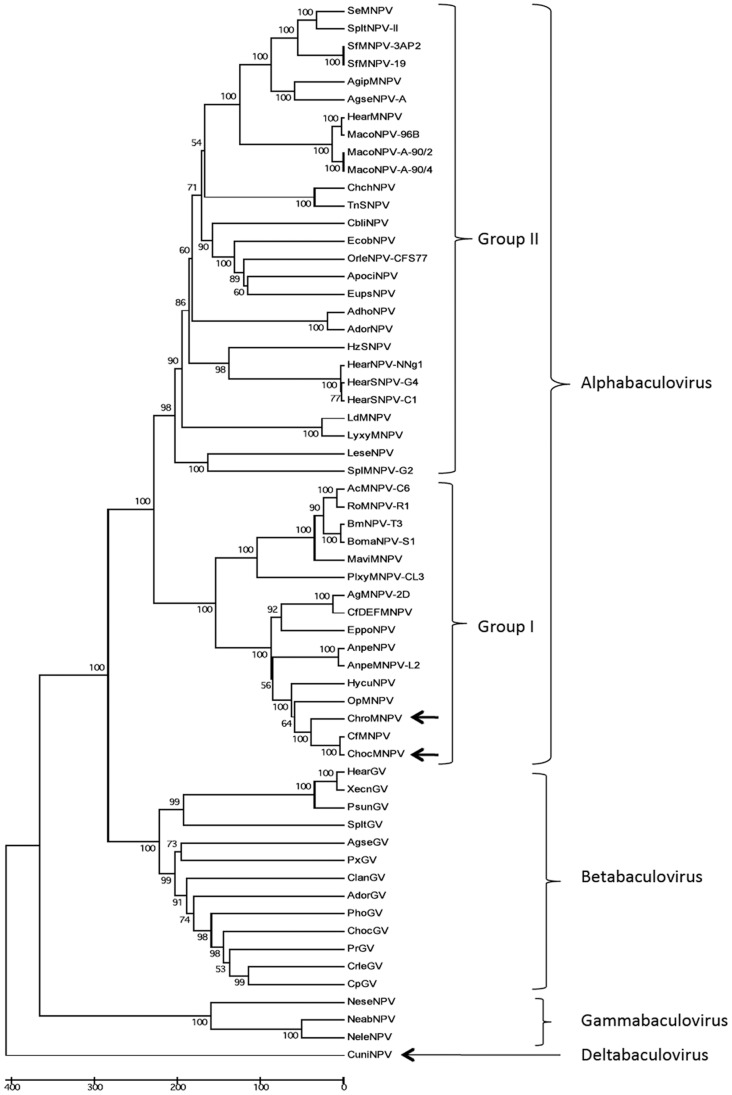
Baculovirus phylogeny. The analysis was based on concatenated amino acid sequence of the *lef-8* and *pif-2* gene products of 59 baculoviruses using MEGA 5 software [41] and a bootstrap of 1000 pseudo-replicates. The tree categorizes baculoviruses according to the current classification scheme [1].

The LEF-8 and PIF-2 concatenated amino acid sequences of both ChocNPV and ChroNPV were compared with their orthologs from the 58 other baculovirus sequences available in the NCBI data base in order to generate a phylogenetic tree. The baculovirus genes *lef-8* and *pif-2*, and their products, have been shown to be the most useful for generating robust trees for inferring baculovirus phylogeny [40]. Consistent with previous studies, the tree separated baculoviruses according to their recent classification scheme [1], with both ChocNPV and ChroNPV being placed in group I alphabaculoviruses (Figure 5). ChocNPV and ChroNPV were clustered together, with ChocNPV being more closely related to CfMNPV than to ChroNPV. These results are also consistent with global alignment and mean amino acid identities of baculovirus homologs. The evolutionary distance among *Choristoneura* NPVs is small, except for CfDEFNPV, which appears to be most closely related to AgMNPV-2D. The more distant relationship of ChroNPV to both ChocNPV and CfMNPV probably relates to the phylogenetic relationship of their respective hosts. SBW and wSBW are both coniferophagous and are more closely related to each other than to OBL, which feeds primarily on members of the Rosaceae [81]. Such co-evolutionary lineages have been demonstrated on a broader scale in other baculoviruses [40]. Based on their high genomic similarities (Tables 1 and S1), the close taxonomic relationship of their respective hosts, and their overlapping geographic distribution [17], CfMNPV and ChocNPV could be considered as variants of the same virus species [82].

In summary, the complete genomes of ChocNPV and ChroNPV alphabaculoviruses were sequenced and compared with each other and other baculoviruses. Both genomes exhibited high sequence similarities to other baculoviruses previously reported in the *Choristoneura* species complex. The ChocNPV genome was more closely related to CfMNPV than ChroNPV. The latter, however, contained a eukaryotic intiation factor 5 (*eiF-5*) homolog that has only been reported in the betabaculovirus ChocGV.

## Supporting Information

Table S1 **Comparison of putative ChocNPV ORFs (left column) with homologous ORFs from five alphabaculoviruses.** ^♦^Nucleotide position of putative ORFs and the orientation of transcription are shown in arrow heads. Homologous regions (*hrs*) are shown in bold underlined characters. The gene names are shown in the second column and italicized. The symbols represent the following; **^†^**ORFs unique to ChocNPV. **^§^**Homologous ORF present in EppoMNPV genome [Eppo ORF28 (53%)]. *Calculation of amino acid identities (%) in homologous ORFs was based on BLASTP.
(DOCX)

Table S2 **Comparison of putative 149 ChroNPV ORFs (left column) with homologous ORFs in five alphabaculoviruses.** ^♦^Nucleotide position of putative ORFs and the orientation of transcription is shown in arrow heads. Homologous regions (*hrs*) are shown in bold underlined characters. The second column represents gene names. The symbols represent the following; **^⊕^**ORFs unique to ChroNPV. **^§^**Homologous ORF present in EppoMNPV genome. *Calculation of amino acid identities (%) in homologous ORFs was based on BLASTP.
(DOCX)
