# Supplementary material for: Comparative Genome Sequence Analysis of Choristoneura occidentalis Freeman and C. rosaceana Harris (Lepidoptera: Tortricidae) Alphabaculoviruses
Source: PLoS One. 2013 Jul 5;8(7):e68968. doi: 10.1371/journal.pone.0068968 (PMC3702617; doi:10.1371/journal.pone.0068968)
Supplement: Table S1 — Comparison of putative ChocNPV ORFs (left column) with homologous ORFs from five alphabaculoviruses. ♦Nucleotide position of putative ORFs and the orientation of transcription are shown in arrow heads. Homologous regions (hrs) are shown in bold underlined characters. The gene names are shown in the second column and italicized. The symbols represent the following; †ORFs unique to ChocNPV. §Homologous ORF present in EppoMNPV genome [Eppo ORF28 (53%)]. *Calculation of amino acid identities (%) in homologous ORFs was based on BLASTP. (DOCX) [file pone.0068968.s001.docx]

**Table S1.** **Comparison of putative ChocNPV ORFs (left column) with homologous ORFs from five alphabaculoviruses.** ^♦^Nucleotide position of putative ORFs and the orientation of transcription are shown in arrow heads. Homologous regions (*hrs*) are shown in bold underlined characters. The gene names are shown in the second column and italicized. The symbols represent the following; **^†^**ORFs unique to ChocNPV. **^§^**Homologous ORF present in EppoMNPV genome [Eppo ORF28 (53%)]. *Calculation of amino acid identities (%) in homologous ORFs was based on BLASTP.

|  |  | | |  |  |  |  |  | | **Baculovirus homologous ORF number (% amino acid ID)^*^** | | | | |  |
| --- | --- | --- | --- | --- | --- | --- | --- | --- | --- | --- | --- | --- | --- | --- | --- |
|  | **ChocNPV ORF number** | | | **Gene name** | **Nucleotide position (bp)^♦^** | **Intergenic distance (bp)** | **Length aa (Da)** | |  | **CfMNPV** | **CfDEFNPV** | **OpMNPV** | **AcMNPV** | **HycuNPV** | |
|  | | 1 | *polh* | | 1 > 738 | 102 | 245 (28764) | |  | 1 (98) | 1 (98) | 3 (99) | 8 (86) | 1(95) | |
|  | | 2 | *1629 cap* | | 735 < 2639 | -4 | 634 (70206) | |  | 146 (98) | 149 (50) | 2 (50) | 9 (29) | 2 (66) | |
|  | | 3 | *pk-1* | | 2638 > 3462 | -2 | 274 (31777) | |  | 145 (99) | 148 (77) | 1 (85) | 10 (69) | 3 (99) | |
|  | | 4 | *pe38* | | 3721 < 4818 | 258 | 365 (42036) | |  | 144 (91) | 146 (44) | 152 (41) | 153 (41) | 4 (38) | |
|  | | 5 |  | | 5129 < 5353 | 310 | 74 (8450) | |  | 143 (39) | - | - | - | - | |
|  | | 6 | *ie-2* | | 5764 > 6858 | 410 | 364 (41766) | |  | 142 (96) | 143 (36) | 151(43) | 151 (31) | 6 (42) | |
|  | | 7 |  | | 7043 > 7261 | 184 | 72 (8652) | |  | 116 (29) | - | - | - | - | |
|  | | **hr5** |  | | 7254 > 7701 |  |  | |  |  |  |  |  |  | |
|  | | 8 | *odv-e56* | | 7769 > 8860 | 507 | 363 (39205) | |  | 141 (92) | 141 (86) | 146 (85) | 148 (54) | 9 (76) | |
|  | | 9 | *ie-1* | | 8933 < 10615 | 72 | 560 (64930) | |  | 140 (99) | 140 (69) | 145 (73) | 147 (56) | 10 (77) | |
|  | | 10 |  | | 10668 > 11261 | 52 | 197 (21964) | |  | 139 (98) | 139 (71) | 144 (73) | 146 (34) | 11 (75) | |
|  | | 11 |  | | 11291 < 11578 | 29 | 95 (10963) | |  | 138 (99) | 138 (84) | 142 (94) | 145 (70) | 12 (88) | |
|  | | 12 | *odv-e27* | | 11581 < 12471 | 2 | 296 (33921) | |  | 137 (100) | 137 (86) | 141 (79) | 144 (71) | 13 (91) | |
|  | | 13 | *odv-e18* | | 12500 < 12757 | 28 | 85 (8972) | |  | 136 (100) | 136 (83) | 140 (92) | 143 (53) | 14 (78) | |
|  | | 14 | *p49* | | 12754 < 14004 | -4 | 416 (48049) | |  | 135 (99) | 135 (92) | 139 (95) | 142 (75) | 15(87) | |
|  | | 15 | *ie-0* | | 14231 < 14965 | 226 | 244 (27294) | |  | 134 (100) | 134 (72) | 138 (73) | 141 (38) | 16 (69) | |
|  | | 16 |  | | 14986 < 15273 | 20 | 95 (11093) | |  | 133 (84) | - | - | - | - | |
|  | | 17 | *me53* | | 15304 > 16650 | 30 | 448 (5187) | |  | 132 (99) | 133 (59) | 137 (78) | 139 (38) | 17 (62) | |
|  | | 18 | *ctl-1* | | 16695 < 16856 | 44 | 53 (5688) | |  | 131 (100) | - | 136 (66) | 3 (81) | 143 (75) | |
|  | | 19 | *p74* | | 16999 > 18936 | 142 | 645 (73134) | |  | 130 (99) | 132 (87) | 134 (90) | 138 (78) | 19 (87) | |
|  | | 20 | *p10* | | 18937 < 19182 | 0 | 81 (8735) | |  | 129 (100) | 131 (55) | 133 (53) | 137 (56) | 20 (88) | |
|  | | 21 | *p26b* | | 19233 < 19934 | 50 | 233 (25742) | |  | 128 (99) | 130 (75) | 132 (66) | 136 (57) | 21 (79) | |
|  | | 22 |  | | 20123 > 20743 | 188 | 206 (23891) | |  | 127 (96) | 129 (59) | - | - | 22 (61) | |
|  | | 23 | *alk-exo* | | 20777 < 22048 | 33 | 423 (47334) | |  | 126 (99) | 128 (69) | 131 (81) | 133 (53) | 23 (76) | |
|  | | 24 |  | | 22063 < 22737 | 14 | 224 (26061) | |  | 125 (97) | 127 (45) | 130 (56) | 132 (40) | 24 (46) | |
|  | | 25 | *pep/calyx* | | 22739 < 23605 | 1 | 288 (31874) | |  | 124 (98) | 126 (72) | 129 (75) | 131 (59) | 25 (87) | |
|  | | 26 | *gp16* | | 23653 < 23961 | 47 | 102 (11529) | |  | 123 (99) | 125 (82) | 128 (83) | 130 (72) | 26 (81) | |
|  | | 27 | *p24* | | 23974 < 24546 | 12 | 190 (21259) | |  | 122 (99) | 124 (76) | 127 (84) | 129 (67) | 27 (77) | |
|  | | 28 | **†** | | 24967 < 25161 | 420 | 64 (7323) | |  | - | - | - | - | - | |
|  | | 29 | *gp64* | | 25337 > 26866 | 175 | 509 (58369) | |  | 119 (99) | 123 (81) | 126 (89) | 128 (80) | 28 (86) | |
|  | | 30 | *v-cath* | | 26934 < 27908 | 67 | 324 (36696) | |  | 118 (99) | 122 (89) | 125 (83) | 127 (79) | 29 (85) | |
|  | | 31 | *v-chi* | | 27952 > 29610 | 43 | 552 (61540) | |  | 117 (99) | 121 (92) | 124 (86) | 126 (33) | 30 (86) | |
|  | | **hr4** |  | | 29640 < 29981 |  |  | |  |  |  |  |  |  | |
|  | | 32 |  | | 29678 > 30028 | 67 | 116 (13397) | |  | 116 (89) | - | - | - | - | |
|  | | 33 | *lef-7* | | 30080 < 30721 | 51 | 213 (24721) | |  | 115 (100) | 120 (67) | 123 (57) | 125 (33) | 31 (54) | |
|  | | 34 | *v-trex* | | 30761 > 31459 | 39 | 232 (25959) | |  | 114 (99) | 119 (67) | - | - | - | |
|  | | 35 |  | | 31835 < 32563 | 375 | 242 (26994) | |  | 113 (99) | 118 (59) | 122 (74) | 124 (44) | 32 (69) | |
|  | | 36 |  | | 32709 > 32909 | 145 | 66 (7193) | |  | 112 (100) | 117 (57) | 121 (54) | 122 (39) | 33 (61) | |
|  | | 37 |  | | 32913 < 33161 | 3 | 82 (9446) | |  | 111 (99) | 115 (60) | 120 (77) | 120 (53) | 34 (62) | |
|  | | 38 | *pif-1* | | 33354 < 34946 | 192 | 530 (58612) | |  | 110 (99) | 114 (84) | 119 (83) | 119 (77) | 35 (86) | |
|  | | 39 |  | | 35055 < 35348 | 108 | 97 (11019) | |  | 109 (99) | 113 (36) | 117 (70) | 117 (33) | 37 (73) | |
|  | | 40 | *bro-d* | | 35378 < 35902 | 29 | 174 (20223) | |  | 107 (97) | 112 (74) | 116 (86) | - | 38 (78) | |
|  | | 41 | *pif-3* | | 35932 > 36549 | 29 | 205 (22502) | |  | 106 (99) | 111 (70) | 115 (78) | 115 (63) | 39 (76) | |
|  | | 42 |  | | 36576 > 37850 | 26 | 424 (48339) | |  | 105 (99) | 109 (54) | 114 (72) | 114 (38) | 40 (68) | |
|  | | 43 |  | | 37866 < 38951 | 15 | 361 (40476) | |  | 104 (100) | 108 (69) | 113(78) | - | 42(76) | |
|  | | 44 |  | | 38887 > 39603 | -65 | 238 (27580) | |  | 103 (99) | 107 (43) | - | - | 43 (58) | |
|  | | 45 |  | | 39613 > 39783 | 9 | 56 (6760) | |  | 101 (100) | 105 (86) | 111 (89) | 110 (71) | 45 (82) | |
|  | | 46 | *odv-ec43* | | 39863 > 41035 | 79 | 390 (44480) | |  | 100 (99) | 103 (84) | 109 (89) | 109 (67) | 46 (87) | |
|  | | 47 |  | | 41038 > 41340 | 2 | 100 (11072) | |  | 99 (100) | 102 (79) | 108 (90) | 108 (56) | 47 (83) | |
|  | | 48 |  | | 41347 < 42114 | 6 | 255 (29453) | |  | 98 (99) | 100 (80) | 107 (77) | 106/107 (71) | 48 (74) | |
|  | | 49 |  | | 42258 > 42446 | 143 | 63 (7071) | |  | - | 98 (71) | - | 105 (80) | - | |
|  | | 50 | *p87* | | 42936 < 44810 | 489 | 624 (71409) | |  | 97 (99) | 96 (52) | 105 (57) | 104 (58) | 49 (51) | |
|  | | 51 | *p48* | | 44834 > 46069 | 23 | 411 (47976) | |  | 96 (100) | 95 (77) | 104 (87) | 103 (56) | 50 (81) | |
|  | | 52 | *p12* | | 46047 > 46382 | -23 | 111 (12008) | |  | 95 (100) | 94 (79) | 103 (85) | 102 (51) | 51 (78) | |
|  | | 53 | *p40* | | 46392 > 47450 | 9 | 352 (39952) | |  | 94 (99) | 93 (78) | 102 (83) | 101 (60) | 52 (84) | |
|  | | 54 | *p6.9* | | 47492 > 47644 | 41 | 50 (6351) | |  | 93 (100) | 92 (87) | 101 (90) | 100 (71) | - | |
|  | | 55 | *lef-5* | | 47641 < 48426 | -4 | 261 (30389) | |  | 92 (100) | 91 (73) | 100 (81) | 99 (59) | 54 (82) | |
|  | | 56 | *p38* | | 48373 > 49302 | -54 | 309 (35941) | |  | 91 (99) | 90 (78) | 99 (82) | 98 (59) | 55 (81) | |
|  | | 57 |  | | 49427 > 50284 | 124 | 285 (34187) | |  | 90 (92) | - | - | - | - | |
|  | | 58 |  | | 50350 > 51255 | 65 | 301 (36934) | |  | 89 (97) | - | - | - | - | |
|  | | 59 | *odv-e28* | | 51252 < 51809 | -4 | 185 (21001) | |  | 88 (100) | 89 (83) | 97 (82) | 96 (69) | 58 (84) | |
|  | | 60 | *hel* | | 51799 > 55485 | -11 | 1228 (141784) | |  | 87 (99) | 88 (73) | 96 (85) | 95 (58) | 59 (83) | |
|  | | 61 | *odv-e25* | | 55800 < 56489 | 314 | 229 (25599) | |  | 86 (99) | 87 (85) | 95 (89) | 94 (63) | 60 (86) | |
|  | | 62 | *p18* | | 56494 < 56973 | 4 | 159 (17968) | |  | 85 (99) | 86 (86) | 94 (94) | 93 (73) | 61 (92) | |
|  | | 63 | *p33* | | 56972 > 57784 | -2 | 270 (32136) | |  | 84 (99) | 85 (80) | 93 (81) | 92 (80) | 62 (82) | |
|  | | 64 |  | | 57800 > 58435 | 15 | 211 (22794) | |  | 83 (99) | - | 92 (71) | 91 (51) | - | |
|  | | 65 | *lef-4* | | 58422 < 59795 | -14 | 457 (51850) | |  | 82 (99) | 83 (70) | 91 (82) | 90 (53) | 64 (82) | |
|  | | 66 | *vp39* | | 59806 > 60861 | 10 | 351 (39483) | |  | 81 (98) | 82 (74) | 90 (91) | 89 (61) | 65 (89) | |
|  | | 67 | *cg30* | | 60867 > 61616 | 5 | 249 (28420) | |  | 80 (99) | 81 (59) | 89 (77) | 88 (46) | 66 (73) | |
|  | | 68 | *capsid-p15* | | 61576 < 61938 | -41 | 120 (14441) | |  | 79 (98) | 80 (38) | 88 (73) | 87 (40) | 67 (73) | |
|  | | **hr3** |  | | 62501 > 62761 |  |  | |  |  |  |  |  |  | |
|  | | 69 |  | | 62568 < 62762 | 629 | 64 (7547) | |  | 116 (48) | - | - | - | - | |
|  | | 70 | *vp91* | | 62851 < 65352 | 88 | 833 (93088) | |  | 78 (99) | 78 (76) | 86 (80) | 83 (61) | 68 (77) | |
|  | | 71 | *tlp* | | 65321 > 65788 | -32 | 155 (17243) | |  | 77 (98) | 77 (59) | 85 (78) | 82 (32) | 69 (75) | |
|  | | 72 |  | | 65670 > 66326 | -119 | 218 (24749) | |  | 76 (99) | 76 (85) | 84 (91) | 81 (72) | 76 (89) | |
|  | | 73 | *gp41* | | 66319 > 67404 | -8 | 361 (39920) | |  | 75 (99) | 75 (83) | 83 (87) | 80 (73) | 71 (87) | |
|  | | 74 |  | | 67408 > 67722 | 3 | 104 (12383) | |  | 74 (100) | 74 (84) | 82 (91) | 79 (69) | 72 (88) | |
|  | | 75 |  | | 67719 > 68039 | -4 | 106 (11944) | |  | 73 (100) | 73 (66) | 81 (72) | 78 (61) | 73(77) | |
|  | | 76 | *vlf-1* | | 68041 > 69165 | 1 | 374 (43208) | |  | 72 (99) | 72(90) | 80 (93) | 77 (83) | 74 (91) | |
|  | | 77 |  | | 69177 > 69431 | 11 | 84 (9326) | |  | 71 (100) | 71 (94) | 79 (95) | 76 (83) | 75 (96) | |
|  | | 78 |  | | 69436 > 69828 | 4 | 130 (14839) | |  | 70 (99) | 70 (55) | 78 (85) | 75 (44) | 76 (82) | |
|  | | 79 |  | | 69841 > 70359 | 12 | 172 (19856) | |  | 69 (98) | 69 (60) | 77 (70) | 74 (44) | 77 (67) | |
|  | | 80 |  | | 70356 > 70601 | -4 | 81 (9417) | |  | 68 (99) | - | 76 (39) | 73 (??) | 78 (39) | |
|  | | 81 |  | | 70632 < 70802 | 30 | 56 (6538) | |  | 67 (100) | 67 (55) | 75 (67) | 72 (53) | 79 (76) | |
|  | | 82 | *iap-2* | | 70835 < 71584 | 32 | 249 (27951) | |  | 66 (96) | 66 (71) | 74 (71) | 71 (58) | 80 (75) | |
|  | | 83 | *met* | | 71565 < 72365 | -20 | 266 (30016) | |  | 65 (100) | 65 (73) | - | 69 (58) | 81 (78) | |
|  | | 84 |  | | 72337 < 72732 | -29 | 131 (15343) | |  | 64 (100) | 64 (85) | 73 (85) | 68 (71) | 82 (87) | |
|  | | 85 | *lef-3* | | 72731 > 73852 | -2 | 373 (42921) | |  | 63 (99) | 63 (62) | 72 (76) | 67 (40) | 83 (74) | |
|  | | 86 | *desmoplakin* | | 73849 < 76467 | -4 | 872 (99943) | |  | 62 (96) | 62 (40) | 71 (56) | 66 (26) | 84 (46) | |
|  | | 87 | *DNApol* | | 76477 > 79449 | 9 | 990 (114302) | |  | 61 (99) | 61 (75) | 70 (84) | 65 (61) | 85 (81) | |
|  | | 88 | *slp* | | 79495 > 80595 | 45 | 366 (42203) | |  | 60 (98) | 60 (82) | 69 (65) | 64 (69) | 86 (67) | |
|  | | 89 | *lef-9* | | 80922 < 82394 | 326 | 490 (55539) | |  | 59 (99) | 58 (89) | 65 (92) | 62 (77) | 89 (91) | |
|  | | 90 | *fp* | | 82458 > 83084 | 63 | 208 (24349) | |  | 58 (98) | 57 (80) | 64 (88) | 61 (71) | 90 (89) | |
|  | | 91 | *chaB* | | 83197 > 83466 | 112 | 89 (9914) | |  | 57 (100) | 56 (68) | 63 (79) | 60 (62) | 91 (82) | |
|  | | 92 |  | | 83438 > 83944 | -29 | 168 (19025) | |  | 56 (99) | 55 (57) | 62 (65) | 59 (41) | 92 (90) | |
|  | | 93 |  | | 83968 < 84450 | 23 | 160 (18317) | |  | 55 (100) | 54 (60) | 61 (77) | 57 (50) | 93 (75) | |
|  | | 94 |  | | 84636 < 84884 | 185 | 82 (9594) | |  | 54 (100) | 53 (64) | 60 (80) | 56 (50) | 94 (77) | |
|  | | 95 |  | | 84886 < 85092 | 1 | 68 (7891) | |  | 53 (97) | 52 (67) | 59 (74) | 55 (55) | 95 (75) | |
|  | | 96 | *vp1054* | | 85154 < 86290 | 61 | 378 (42886) | |  | 52 (99) | 51 (72) | 58 (84) | 54 (52) | 96 (79) | |
|  | | 97 | *lef-10* | | 86139 < 86381 | -152 | 80 (8389) | |  | 51 (100) | 50 (73) | 57 (73) | 53a (44) | 97 (83) | |
|  | | 98 |  | | 86350 < 86790 | -32 | 146 (16952) | |  | 50 (100) | 49 (82) | 56 (84) | 53 (58) | 98 (92) | |
|  | | 99 | *bjdp* | | 86919 < 87854 | 128 | 311 (36161) | |  | 49 (99) | 48 (46) | 55 (55) | 51(31) | 101 (52) | |
|  | | 100 | *lef-8* | | 87887 > 90508 | 32 | 873 (99693) | |  | 48 (99) | 47 (82) | 54 (87) | 50 (70) | 102 (87) | |
|  | | 101 | *pcna* | | 90539 > 91273 | 30 | 244 (26553) | |  | 47 (98) | - | 53 (64) | 49 (35) | 103 (64) | |
|  | | 102 | *etm* | | 91287 > 91664 | 13 | 25 (13891) | |  | 46 (98) | 46 (70) | 52 (79) | 48 (42) | 104 (77) | |
|  | | 103 | *odv-e66* | | 91735 < 93771 | 70 | 678 (75643) | |  | 45 (96) | 45 (82) | 50 (78) | 46 (76) | 106 (76) | |
|  | | 104 |  | | 93823 < 94188 | 51 | 121 (13337) | |  | 44 (100) | 44 (70) | 49 (65) | 44 (47) | - | |
|  | | 105 |  | | 94151 < 94351 | -38 | 66 (7578) | |  | 43 (78) | 43 (80) | 48 (81) | 43 (60) | 107 (79) | |
|  | | 106 | *gta* | | 94354 < 95847 | 2 | 497 (57587) | |  | 42 (99) | 42 (77) | 47 (87) | 42 (60) | 108 (86) | |
|  | | 107 | *lef-12* | | 95856 < 96770 | 8 | 304 (33502) | |  | 41 (99) | 41 (70) | 46 (79) | 41 (46) | 109 (73) | |
|  | | 108 | *p47* | | 96355 > 97554 | -416 | 399 (46340) | |  | 40 (99) | 40 (82) | 45 (83) | 40 (68) | 110 (84) | |
|  | | 109 | *pkip* | | 97608 < 98108 | 53 | 166 (18624) | |  | 39 (99) | 39 (65) | 44 (80) | 24 (48) | 111 (73) | |
|  | | 110 | *ssdbp* | | 98118 < 99020 | 9 | 300 (34609) | |  | 38 (99) | 38 (70) | 43 (89) | 25 (42) | 112 (68) | |
|  | | 111 |  | | 99075 > 99452 | 54 | 125 (14142) | |  | 37 (98) | 37 (69) | 42 (78) | 26 (60) | 113 (79) | |
|  | | 112 | *iap-1* | | 99449 > 100279 | -4 | 276 (31327) | |  | 36 (99) | 36 (70) | 41 (84) | 27 (56) | 114 (79) | |
|  | | 113 | *lef-6* | | 100279 > 100653 | -1 | 124 (14757) | |  | 35 (99) | 35 (57) | 40 (62) | 28 (27) | 115 (65) | |
|  | | 114 |  | | 100701 < 100907 | 47 | 68 (8056) | |  | 34 (99) | 34 (67) | 39 (66) | 29 (56) | 116 (68) | |
|  | | 115 |  | | 100962 < 102335 | 54 | 457 (52916) | |  | 33 (99) | 33 (67) | 38 (78) | 30 (52) | 117 (74) | |
|  | | 116 |  | | 102334 > 102897 | -2 | 187 (21040) | |  | 32 (100) | 32 (46) | 37 (61) | - | 118 (58) | |
|  | | 117 |  | | 102929 < 103624 | 31 | 231 (24201) | |  | 31 (97) | 31 (50) | 36 (69) | - | 119 (60) | |
|  | | 118 | *iap-3* | | 103704 > 104612 | 79 | 302 (34461) | |  | 30 (70) | 30 (53) | 35 (51) | - | 120 (53) | |
|  | | **hr2** |  | | 104768 < 105142 |  |  | |  |  |  |  |  |  | |
|  | | 119 | **†** | | 104872 < 105180 | 259 | 102 (10812) | |  | - | - | - | - | - | |
|  | | 120 | *vef* | | 105268 > 107556 | 87 | 762 (87120) | |  | 29 (99) | - | - | - | - | |
|  | | 121 | *sod* | | 107563 > 108021 | 6 | 152 (15944) | |  | 28 (99) | 29 (85) | 29 (82) | 31 (77) | 122 (83) | |
|  | | 122 | **§** | | 108085 < 108252 | 63 | 55 (6580) | |  | - | - | - | - | - | |
|  | | 123 | *fgf* | | 108408 < 108947 | 155 | 179 (20349) | |  | 27 (97) | 28 (64) | 27 (72) | 32 (41) | 124 (65) | |
|  | | **hr1** |  | | 109054 > 109460 |  |  | |  |  |  |  |  |  | |
|  | | 124 |  | | 109473 < 110099 | 525 | 208 (24278) | |  | 26 (99) | 26 (72) | 26 (80) | 34 (53) | 125 (74) | |
|  | | 125 | *v-ubi* | | 110059 > 110352 | -41 | 94 (10714) | |  | 25 (100) | 25 (95) | 25 (97) | 35 (88) | 126 (95) | |
|  | | 126 | *39k* | | 110385 < 111167 | 32 | 260 (29693) | |  | 24 (99) | 24 (75) | 24 (80) | 36 (50) | 127 (81) | |
|  | | 127 | *lef-11* | | 111161 < 111484 | -7 | 107 (12410) | |  | 23 (99) | 23 (73) | 23 (82) | 37 (53) | 128 (83) | |
|  | | 128 |  | | 111492 < 112097 | 7 | 201 (23528) | |  | 22 (100) | 22 (86) | 22 (92) | 38 (77) | 129 (89) | |
|  | | 129 | *f-protein* | | 112223 < 114187 | 125 | 654 (73522) | |  | 21 (99) | 21 (57) | 21 (70) | 23 (38) | 130 (64) | |
|  | | 130 | *pif-2* | | 114339 < 115490 | 151 | 383 (43752) | |  | 20 (100) | 10 (90) | 20 (90) | 22 (79) | 131 (89) | |
|  | | 131 | *arif-1* | | 115515 > 116555 | 24 | 346 (39516) | |  | 19 (97) | 11 (61) | 19 (70) | 21 (39) | 132( 54) | |
|  | | 132 |  | | 116657 < 116971 | 101 | 104 (11761) | |  | 18 (100) | 12 (72) | 18 (70) | 19 (44) | 133 (74) | |
|  | | 133 |  | | 116984 > 118042 | 12 | 352 (40127) | |  | 17 (100) | 13 (63) | 17 (74) | 18 (51) | 134 (76) | |
|  | | 134 |  | | 118131 < 118754 | 88 | 207 (23063) | |  | 16 (99) | 14 (71) | 16 (72) | 17 (49) | 135 (66) | |
|  | | 135 | *odv-e26* | | 118723 < 119325 | -32 | 200 (22212) | |  | 15 (98) | 15 (59) | 15 (72) | 16 (34) | 137 (72) | |
|  | | 136 | *egt* | | 119481 < 120953 | 155 | 490 (54378) | |  | 14 (99) | 16 (74) | 14 (81) | 15 (64) | 138 (78) | |
|  | | 137 | *lef-1* | | 121022 > 121762 | 68 | 246 (28006) | |  | 13 (100) | 17 (68) | 13 (74) | 14 (57) | 139 (78) | |
|  | | 138 |  | | 121705 > 122661 | -58 | 318 (36571) | |  | 12 (98) | 18 (70) | 12 (74) | 13 (46) | 140 (72) | |
|  | | 139 |  | | 122704 > 123345 | 42 | 213 (23858) | |  | 11 (99) | 20 (47) | - | - | - | |
|  | | 140 |  | | 123383 < 124396 | 37 | 337 (38652) | |  | 10 (98) | 9 (55) | 11 (69) | 11 (41) | 141 (58) | |
|  | | 141 | *ptp-1* | | 124465 > 124998 | 68 | 177 (20460) | |  | 09 (99) | 8 (72) | 10 (79) | 1 (66) | 142 (72) | |
|  | | 142 | *ptp-2* | | 124976 > 125458 | -23 | 160 (18257) | |  | 08 (100) | 7 (74) | 9 (72) | - | - | |
|  | | 143 | *p26a* | | 125496 > 126299 | 37 | 267 (30107) | |  | 07 (99) | 130 (32) | 132 (32) | 136 (35) | 21 (32) | |
|  | | 144 | *bro* | | 126378 < 126581 | 78 | 67 (8020) | |  | 06 (86) | - | - | - | - | |
|  | | 145 |  | | 126618 < 127049 | 36 | 143 (16064) | |  | 05 (94) | 5 (77) | 8 (73) | 4 (61) | 145 (78) | |
|  | | 146 |  | | 127094 > 127324 | 44 | 76 (8415) | |  | 04 (99) | 4 (61) | 7 (83) | 5 (60) | 146 (60) | |
|  | | 147 | *lef-2* | | 127327 > 127935 | 2 | 202 (22572) | |  | 03 (99) | 3 (71) | 6 (81) | 6 (56) | 147 (78) | |
|  | | 148 |  | | 127958 > 128344 | 22 | 128 (14625) | |  | 02 (95) | 2 (62) | 5 (78) | - | - | |
|  | |  |  | |  |  |  | |  |  |  |  |  |  | |
