# Supplementary material for: Comparative Genome Sequence Analysis of Choristoneura occidentalis Freeman and C. rosaceana Harris (Lepidoptera: Tortricidae) Alphabaculoviruses
Source: PLoS One. 2013 Jul 5;8(7):e68968. doi: 10.1371/journal.pone.0068968 (PMC3702617; doi:10.1371/journal.pone.0068968)
Supplement: Table S2 — Comparison of putative 149 ChroNPV ORFs (left column) with homologous ORFs in five alphabaculoviruses. ♦Nucleotide position of putative ORFs and the orientation of transcription is shown in arrow heads. Homologous regions (hrs) are shown in bold underlined characters. The second column represents gene names. The symbols represent the following; ⊕ORFs unique to ChroNPV. §Homologous ORF present in EppoMNPV genome. *Calculation of amino acid identities (%) in homologous ORFs was based on BLASTP. (DOCX) [file pone.0068968.s002.docx]

**Table S2.** **Comparison of putative 149 ChroNPV ORFs (left column) with homologous ORFs in five alphabaculoviruses.** ^♦^Nucleotide position of putative ORFs and the orientation of transcription is shown in arrow heads. Homologous regions (*hrs*) are shown in bold underlined characters. The second column represents gene names. The symbols represent the following; **^⊕^**ORFs unique to ChroNPV. **^§^**Homologous ORF present in EppoMNPV genome. *Calculation of amino acid identities (%) in homologous ORFs was based on BLASTP.

|  |  |  |  |  |  |  | **Baculovirus homologous ORF # (% amino acid ID)^*^** | | | | |
| --- | --- | --- | --- | --- | --- | --- | --- | --- | --- | --- | --- |
|  | **ChocNPV ORF number** | **Gene name** | **Nucleotide position (bp)^♦^** | **Intergenic distance (bp)** | **Length aa (Da)** |  | **CfMNPV** | **CfDEFNPV** | **OpMNPV** | **AcMNPV** | **HycuNPV** |
|  | 1 | *polh* | 1 > 738 | 98 | 245 (28788) |  | 1 (98) | 1 (98) | 3 (99) | 8 (89) | 1 (96) |
|  | 2 | *1629 cap* | 735 < 2621 | -4 | 628 (69474) |  | 146 (79) | 149 (47) | 2 (50) | 9 (46) | 2 (66) |
|  | 3 | *pk-1* | 2623 > 3447 | 1 | 274 (31683) |  | 145 (91) | 148 (77) | 1 (85) | 10 (67) | 3 (85) |
|  | 4 |  | 3438 < 3665 | -10 | 75 (8113) |  | - | 147 (43) | - | - | 4 (39) |
|  | 5 | *pe38* | 3796 < 4959 | 130 | 387 (44898) |  | 144 (63) | 146 (49) | 152 (38) | 153 (37) | 5 (36) |
|  | 6 | *ie-2* | 5553 > 6941 | 593 | 362 (53122) |  | 142 (61) | 143 (38) | 151(36) | 151 (31) | 6 (42) |
|  | 7 | **^⊕^** | 6996 > 7250 | 54 | 84 (10121) |  | - | - | - | - | - |
|  | 8 | **^⊕^** | 7323 < 7511 | 72 | 62 (7365) |  | - | - | - | - | - |
|  | 9 | *odv-e56* | 7653 > 8789 | 141 | 378 (40549) |  | 141 (97) | 141 (90) | 146 (89) | 148 (70) | 9 (82) |
|  | 10 | *ie-1* | 8930 < 10675 | 140 | 581(67187) |  | 140 (85) | 140 (71) | 145 (76) | 147 (52) | 10 (82) |
|  | 11 |  | 10674 > 11273 | -2 | 199 (22464) |  | 139 (84) | 139 (70) | 144 (75) | 146 (51) | 11 (74) |
|  | 12 |  | 11307 < 11594 | 33 | 95 (10969) |  | 138 (95) | 138 (82) | 142 (93) | 145 (71) | 12 (91) |
|  | 13 | *odv-e27* | 11598 < 12491 | 3 | 297 (34055) |  | 137 (95) | 137 (86) | 141 (80) | 144 (69) | 13 (86) |
|  | 14 | *odv-e18* | 12520 < 12777 | 28 | 85 (9059) |  | 136 (93) | 136 (83) | 140 (87) | 143 (68) | 14 (74) |
|  | 15 | *p49* | 12783 < 14234 | 5 | 483 (55951) |  | 135 (94) | 135 (91) | 139 (93) | 142 (74) | 15(85) |
|  | 16 | *ie-0* | 14247 < 14969 | 12 | 240 (26686) |  | 134 (85) | 134 (76) | 138 (76) | 141 (62) | 16 (72) |
|  | 17 | *me53* | 15235 >16593 | 265 | 452 (52214) |  | 132 (77) | 133 (57) | 137 (73) | 139 (38) | 17 (72) |
|  | 18 | *ctl-1* | 16629 < 16790 | 35 | 53 (5618) |  | 131 (91) | - | 136 (74) | 3 (87) | 143 (81) |
|  | 19 |  | 16870 < 17637 | 79 | 255 (29742) |  | - | - | 135 (59) | - | 18 (60) |
|  | 20 | *p74* | 17907 > 19844 | 269 | 645 (73264) |  | 130 (92) | 132 (87) | 134 (89) | 138 (78) | 19 (87) |
|  | 21 | *p10* | 19850 < 20095 | 5 | 81 (8754) |  | 129 (93) | 131 (53) | 133 (52) | 137 (57) | 20 (84) |
|  | 22 | *p26b* | 20146 < 20847 | 50 | 233 (25801) |  | 128 (87) | 130 (72) | 132 (67) | 136 (55) | 21 (78) |
|  | 23 |  | 21076 > 21720 | 228 | 214 (25000) |  | 127 (70) | 129 (59) | - | - | 22 (59) |
|  | 24 | *alk-exo* | 21762 < 23015 | 41 | 417 (46655) |  | 126 (86) | 128 (72) | 131 (82) | 133 (53) | 23 (78) |
|  | 25 |  | 23030 < 23659 | 14 | 209 (24650) |  | 125 (59) | 127 (41) | 130 (53) | 132 (37) | 24 (49) |
|  | 26 | *pep/calyx* | 23661 < 24530 | 1 | 289 (32023) |  | 124 (96) | 126 (72) | 129 (75) | 131 (59) | 25 (88) |
|  | 27 | *gp16* | 24578 < 24886 | 47 | 102 (11488) |  | 123 (87) | 125 (81) | 128 (83) | 130 (73) | 26 (81) |
|  | 28 | *p24* | 24899 < 25477 | 12 | 192 (21554) |  | 122 (90) | 124 (76) | 127 (81) | 129 (67) | 27 (76) |
|  | 29 | *gp64* | 25760 > 27289 | 282 | 509 (58339) |  | 119 (92) | 123 (80) | 126 (86) | 128 (78) | 28 (84) |
|  | 30 | *v-cath* | 27335 < 28309 | 45 | 324 (36881) |  | 118 (92) | 122 (89) | 125 (81) | 127 (79) | 29 (88) |
|  | 31 | *v-chi* | 28353 > 30011 | 43 | 552 (61708) |  | 117 (93) | 121 (91) | 124 (84) | 126 (78) | 30 (84) |
|  | 32 | **^⊕^** | 30037 > 30249 | 25 | 70 (7537) |  | - | - | - | - | - |
|  | **hr4** |  | 30077 > 31016 |  |  |  |  |  |  |  |  |
|  | 33 |  | 30258 > 30458 | 8 | 66 (7766) |  | 116 (41) | - | - | - | - |
|  | 34 | *lef-7* | 30525 >31754 | 66 | 409 (48354) |  | 115 (65) | 120 (61) | 123 (56) | 125 (29) | 31 (53) |
|  | 35 |  | 31897 < 32628 | 142 | 243 (27073) |  | 113 (74) | 118 (55) | 122 (71) | 124 (40) | 32 (69) |
|  | 36 |  | 32677 > 32967 | 48 | 96 (10829) |  | 112 (49) | 117 (47) | 121 (47) | 122 (36) | 33 (49) |
|  | 37 |  | 32968 < 33258 | 0 | 96 (11283) |  | 111 (77) | 115 (59) | 120 (73) | 120 (51) | 34 (65) |
|  | 38 | **^§^** *eif-5* | 33288 < 34643 | 29 | 451 (50251) |  | - | - | - | - | - |
|  | 39 | *pif-1* | 34800 < 36392 | 156 | 530 (59077) |  | 110 (87) | 114 (82) | 119 (81) | 119 (74) | 35 (83) |
|  | 40 |  | 36596 < 36889 | 203 | 97 (11242) |  | 109 (74) | 113 (37) | 117 (66) | 117 (34) | 37 (69) |
|  | 41 | *bro-d* | 36919 < 37443 | 29 | 174 (20276) |  | 107 (77) | 112 (75) | 116 (84) | - | 38 (84) |
|  | 42 | *pif-3* | 37473 > 38090 | 29 | 205 (22502) |  | 106 (85) | 111 (75) | 115 (82) | 115 (66) | 39 (80) |
|  | 43 |  | 38117 > 39409 | 26 | 430 (50196) |  | 105 (75) | 109 (50) | 114 (64) | 114 (39) | 40 (63) |
|  | 44 |  | 39598 > 39810 | 188 | 70 (8011) |  | 102 (80) | 106 (70) | 112 (71) | 111 (62) | 44 (70) |
|  | 45 |  | 39872 > 40042 | 61 | 56 (6754) |  | 101 (95) | 105 (91) | 111 (93) | 110 (75) | 45 (88) |
|  | 46 | *odv-ec43* | 40127 > 41293 | 84 | 388 (44476) |  | 100 (95) | 103 (84) | 109 (89) | 109 (67) | 46 (87) |
|  | 47 |  | 41296 > 41598 | 2 | 100 (11040) |  | 99 (86) | 102 (76) | 108 (84) | 108 (59) | 47 (78) |
|  | 48 |  | 41613 < 42383 | 14 | 256 (29328) |  | 98 (86) | 100 (77) | 107 (77) | 106/107 (71) | 48 (74) |
|  | 49 | *p87* | 42744 < 44522 | 360 | 592 (68278) |  | 97 (55) | 96 (52) | 105 (53) | 104 (58) | 49 (55) |
|  | 50 | *p48* | 44546 > 45781 | 23 | 411 (48199) |  | 96 (91) | 95 (78) | 104 (86) | 103 (57) | 50 (81) |
|  | 51 | *p12* | 45759 > 46094 | -23 | 111 (12040) |  | 95 (95) | 94 (75) | 103 (82) | 102 (41) | 51 (75) |
|  | 52 | *p40* | 46112 > 47167 | 17 | 351 (39804) |  | 94 (89) | 93 (78) | 102 (84) | 101 (60) | 52 (84) |
|  | 53 | *p6.9* | 47209 > 47364 | 41 | 51 (6359) |  | 93 (90) | 92 (90) | 101 (90) | 100 (74) | - |
|  | 54 | *lef-5* | 47361 < 48155 | -4 | 264 (30353) |  | 92 (89) | 91 (74) | 100 (80) | 99 (59) | 54 (80) |
|  | 55 | *p38* | 48102 > 49031 | -54 | 309 (35934) |  | 91 (88) | 90 (79) | 99 (84) | 98 (58) | 55 (82) |
|  | 56 |  | 49184 > 49990 | 152 | 268 (32633) |  | 90 (64) | - | - | - | - |
|  | 57 |  | 50147 > 51046 | 156 | 299 (36934) |  | 89 (61) | - | - | - | - |
|  | 58 | *odv-e28* | 51066 < 51608 | 19 | 180 (20454) |  | 88 (88) | 89 (82) | 97 (81) | 96 (71) | 58 (84) |
|  | 59 | *hel* | 51586 > 55278 | -23 | 1230(142046) |  | 87 (88) | 88 (76) | 96 (89) | 95 (59) | 59 (86) |
|  | 60 | *odv-e25* | 55333 < 56022 | 54 | 229 (25580) |  | 86 (96) | 87 (87) | 95 (90) | 94 (64) | 60 (88) |
|  | 61 | *p18* | 56027 < 56506 | 4 | 159 (18011) |  | 85 (99) | 86 (86) | 94 (94) | 93 (74) | 61 (93) |
|  | 62 | *p33* | 56505 > 57317 | -2 | 270 (32241) |  | 84 (93) | 85 (81) | 93 (81) | 92 (81) | 62 (84) |
|  | 63 |  | 57334 > 58083 | 16 | 249 (26694) |  | 83 (87) | - | 92 (71) | 91 (56) | 63 (74) |
|  | 64 | *lef-4* | 58070 < 59443 | -14 | 457 (51976) |  | 82 (89) | 83 (71) | 91 (83) | 90 (53) | 64 (83) |
|  | 65 | *vp39* | 59455 > 60537 | 11 | 360 (40818) |  | 81 (84) | 82 (73) | 90 (85) | 89 (62) | 65 (83) |
|  | 66 | *cg30* | 60543 > 61361 | 5 | 272 (31656) |  | 80 (76) | 81 (59) | 89 (72) | 88 (48) | 66 (71) |
|  | 67 | **^⊕^** | 61481 > 61723 | 119 | 80 (9307) |  | - | - | - | - | - |
|  | 68 | *vp91* | 61791 < 64265 | 67 | 824 (93014) |  | 78 (84) | 78 (77) | 86 (81) | 83 (62) | 68 (78) |
|  | 69 | *tlp* | 64234 > 64701 | -32 | 155 (17165) |  | 77 (85) | 77 (58) | 85 (81) | 82 (31) | 69 (77) |
|  | 70 |  | 64583 > 65239 | -119 | 218 (24856) |  | 76 (91) | 76 (83) | 84 (89) | 81 (83) | 76 (87) |
|  | 71 | *gp41* | 65232 > 66320 | -8 | 362 (40040) |  | 75 (93) | 75 (84) | 83 (86) | 80 (71) | 71 (88) |
|  | 72 |  | 66324 > 66638 | 3 | 104 (12296) |  | 74 (95) | 74 (82) | 82 (90) | 79 (68) | 72 (88) |
|  | 73 |  | 66635 > 66961 | -4 | 108 (12417) |  | 73 (83) | 73 (70) | 81 (71) | 78 (62) | 73 (73) |
|  | 74 | *vlf-1* | 66963 > 68099 | 1 | 378 (43669) |  | 72 (94) | 72 (89) | 80 (93) | 77 (82) | 74 (90) |
|  | 75 |  | 68111 > 68365 | 11 | 84 (9296) |  | 71 (99) | 71 (95) | 79 (96) | 76 (82) | 75 (98) |
|  | 76 |  | 68370 > 68762 | 4 | 130 (14950) |  | 70 (85) | 70 (57) | 78 (83) | 75 (42) | 76 (85) |
|  | 77 |  | 68775 > 69302 | 12 | 175 (19944) |  | 69 (83) | 69 (63) | 77 (83) | 74 (44) | 77 (68) |
|  | 78 |  | 69299 > 69544 | -4 | 81 (9344) |  | 68 (47) | - | 76 (41) | 73 (31) | 78 (40) |
|  | 79 |  | 69561 < 69731 | 16 | 56 (6326) |  | 67 (83) | 67 (61) | 75 (80) | 72 (59) | 79 (81) |
|  | 80 | *iap-2* | 69764 < 70486 | 32 | 240 (27185) |  | 66 (79) | 66 (71) | 74 (71) | 71 (59) | 80 (73) |
|  | 81 | *met* | 70467 < 71270 | -20 | 267 (29827) |  | 65 (85) | 65 (72) | - | 69 (58) | 81 (75) |
|  | 82 |  | 71242 < 71637 | -29 | 131 (15393) |  | 64 (88) | 64 (81) | 73 (86) | 68 (71) | 82 (81) |
|  | 83 | *lef-3* | 71639 > 72757 | -2 | 372 (43001) |  | 63 (83) | 63 (63) | 72 (79) | 67 (39) | 83 (78) |
|  | 84 | *desmoplakin* | 72754 < 75309 | -4 | 851 (98039) |  | 62 (71) | 62 (40) | 71 (51) | 66 (43) | 84 (46) |
|  | 85 | *DNApol* | 75319 > 78288 | 9 | 989 (113917) |  | 61 (86) | 61 (76) | 70 (82) | 65 (61) | 85 (80) |
|  | 86 | *slp* | 78321 > 79406 | 32 | 361 (41227) |  | 60 (83) | 60 (88) | 69 (68) | 64 (68) | 86 (70) |
|  | 87 | *bro-b* | 79464 > 80429 | 57 | 321 (36024) |  | - | 59 (95) | - | - | 88 (66) |
|  | 88 | *lef-9* | 80470 < 81942 | 40 | 490 (55704) |  | 59 (95) | 58 (89) | 65 (92) | 62 (76) | 89 (92) |
|  | 89 | *fp* | 82011 > 82637 | 68 | 208 (24368) |  | 58 (93) | 57 (82) | 64 (91) | 61 (71) | 90 (91) |
|  | 90 | *chaB* | 82790 > 83059 | 152 | 89 (10045) |  | 57 (83) | 56 (68) | 63 (80) | 60 (64) | 91 (65) |
|  | 91 |  | 83031 > 83516 | -29 | 161 (18554) |  | 56 (68) | 55 (58) | 62 (66) | 59 (45) | 92 (65) |
|  | 92 |  | 83551 < 84123 | 34 | 190 (22075) |  | 55 (87) | 54 (63) | 61 (80) | 57 (53) | 93 (78) |
|  | 93 |  | 84225 < 84476 | 101 | 83 (9712) |  | 54 (84) | 53 (64) | 60 (82) | 56 (47) | 94 (78) |
|  | 94 |  | 84478 < 84684 | 1 | 68 (7925) |  | 53 (89) | 52 (68) | 59 (79) | 55 (58) | 95 (79) |
|  | 95 | *vp1054* | 84746 < 85891 | 61 | 381 (43274) |  | 52 (91) | 51 (73) | 58 (83) | 54 (52) | 96 (81) |
|  | 96 | *lef-10* | 85740 < 85982 | -152 | 80 (8456) |  | 51 (90) | 50 (69) | 57 (66) | 53a (45) | 97 (79) |
|  | 97 |  | 85951 < 86391 | -32 | 146 (16976) |  | 50 (90) | 49 (79) | 56 (83) | 53 (63) | 98 (82) |
|  | 98 | *bjdp* | 86501 < 87424 | 109 | 307 (36349) |  | 49 (49) | 48 (44) | 55 (44) | 51(31) | 101 (50) |
|  | 99 | *lef-8* | 87457 > 90078 | 32 | 873 (99546) |  | 48 (92) | 47 (83) | 54 (88) | 50 (70) | 102 (88) |
|  | 100 | *pcna* | 90114 > 90869 | 35 | 251 (27637) |  | 47 (86) | - | 53 (65) | 49 (36) | 103 (68) |
|  | 101 | *etm* | 90871 > 91203 | 1 | 110 (12419) |  | 46 (89) | 46 (75) | 52 (76) | 48 (43) | 104 (73) |
|  | 102 | *odv-e66* | 91337 < 93376 | 133 | 679 (76011) |  | 45 (90) | 45 (83) | 50 (79) | 46 (78) | 106 (78) |
|  | 103 |  | 93433 < 93801 | 56 | 122 (13501) |  | 44 (85) | 44 (69) | 49 (63) | 44 (47) | - |
|  | 104 |  | 93764 < 93964 | -38 | 66 (7747) |  | 43 (87) | 43 (89) | 48 (76) | 43 (67) | 107 (72) |
|  | 105 | *gta* | 93967 < 95466 | 2 | 499 (57780) |  | 42 (92) | 42 (76) | 47 (86) | 42 (60) | 108 (87) |
|  | 106 | *lef-12* | 95475 < 96011 | 8 | 178 (19718) |  | 41 (86) | 41 (75) | 46 (79) | 41 (49) | 109 (75) |
|  | 107 | *p47* | 95974 > 97179 | -38 | 401 (46570) |  | 40 (90) | 40 (82) | 45 (82) | 40 (69) | 110 (85) |
|  | 108 | *pkip* | 97276 < 97776 | 96 | 166 (18518) |  | 39 (87) | 39 (65) | 44 (85) | 24 (46) | 111 (78) |
|  | 109 | *ssdbp* | 97789 < 98691 | 12 | 300 (34484) |  | 38 (94) | 38 (70) | 43 (88) | 25 (42) | 112 (68) |
|  | 110 |  | 98738 > 99115 | 46 | 125 (14120) |  | 37 (90) | 37 (69) | 42 (79) | 26 (58) | 113 (78) |
|  | 111 | *iap-1* | 99112 > 99945 | -4 | 277 (31381) |  | 36 (84) | 36 (73) | 41 (86) | 27 (58) | 114 (82) |
|  | 112 | *lef-6* | 99945 > 100337 | -1 | 130 (15368) |  | 35 (73) | 35 (52) | 40 (64) | 28 (30) | 115 (67) |
|  | 113 |  | 100376 < 100591 | 38 | 71 (8660) |  | 34 (78) | 34 (78) | 39 (64) | 29 (53) | 116 (72) |
|  | 114 |  | 100630 < 102009 | 38 | 459 (53471) |  | 33 (82) | 33 (68) | 38 (77) | 30 (50) | 117 (75) |
|  | 115 |  | 101984 > 102457 | -26 | 187 (18049) |  | 32 (36) | 32 (48) | 37 (50) | - | 118 (33) |
|  | 116 |  | 102454 < 103122 | -4 | 222 (23694) |  | 31 (73) | 31 (50) | 36 (65) | - | 119 (60) |
|  | 117 | *iap-3* | 103208 > 104146 | 85 | 312 (35134) |  | 30 (69) | 30 (53) | 35 (65) | - | 120 (54) |
|  | 118 |  | 104226 > 104588 | 79 | 120 (14234) |  | 116 (25) | - | - | - | - |
|  | **hr2** |  | 104304 <104515 |  |  |  |  |  |  |  |  |
|  | 119 | *ctl-2* | 104531 < 104689 | -58 | 52 (5606) |  | - | - | 30 (85) | - | 123 (79) |
|  | 120 | *vef* | 104761 > 107043 | 71 | 760 (86341) |  | 29 (85) | - | - | - | - |
|  | 121 | *sod* | 107060 > 107518 | 16 | 152 (15903) |  | 28 (91) | 29 (89) | 29 (81) | 31 (73) | 122 (80) |
|  | 122 | **§** | 107640 < 107906 | 121 | 88 (10821) |  | - | - | - | - | - |
|  | 123 | *fgf* | 107977 < 108573 | 70 | 198 (22977) |  | 27 (77) | 28 (66) | 27 (75) | 32 (43) | 124 (68) |
|  | 124 |  | 108726 < 108983 | 152 | 85 (10089) |  | 116 (33) | - | - | - | - |
|  | **hr1** |  | 108737 > 109602 |  |  |  |  |  |  |  |  |
|  | 125 |  | 109053 < 109307 | 69 | 84 (10011) |  | 116 (39) | - | - | - | - |
|  | 126 |  | 109627 < 110256 | 319 | 209 (24463) |  | 26(81) | 26 (71) | 26 (76) | 34 (51) | 125 (73) |
|  | 127 | *v-ubi* | 110222 > 110512 | -35 | 96 (10656) |  | 25 (96) | 25 (92) | 25 (96) | 35 (87) | 126 (94) |
|  | 128 | *39k* | 110550 < 111332 | 37 | 260 (29803) |  | 24 (86) | 24 (78) | 24 (86) | 36 (53) | 127 (89) |
|  | 129 | *lef-11* | 111272 < 111649 | -61 | 125 (14275) |  | 23 (86) | 23 (72) | 23 (74) | 37 (50) | 128 (81) |
|  | 130 |  | 111636 < 112262 | -14 | 208 (24101) |  | 22 (100) | 22 (86) | 22 (91) | 38 (76) | 129 (88) |
|  | 131 | *f-protein* | 112371 < 114392 | 108 | 673 (76407) |  | 21 (69) | 21 (51) | 21 (63) | 23 (35) | 130 (58) |
|  | 132 | *pif-2* | 114500 < 115648 | 107 | 382 (43369) |  | 20 (93) | 10 (89) | 20 (90) | 22 (78) | 131 (88) |
|  | 133 | *arif-1* | 115673 > 116857 | 24 | 394 (44674) |  | 19 (72) | 11 (55) | 19 (66) | 21 (37) | 132 (51) |
|  | 134 |  | 116984 < 117298 | 126 | 104 (11765) |  | 18 (83) | 12 (65) | 18 (67) | 19 (45) | 133 (68) |
|  | 135 |  | 117311 > 118366 | 12 | 351 (39939) |  | 17 (88) | 13 (63) | 17 (71) | 18 (48) | 134 (75) |
|  | 136 |  | 118811 < 119434 | 444 | 207 (23126) |  | 16 (86) | 14 (75) | 16 (74) | 17 (50) | 135 (73) |
|  | 137 | *odv-e26* | 119403 < 120002 | -32 | 199 (22406) |  | 15 (75) | 15 (57) | 15 (66) | 16 (34) | 137 (66) |
|  | 138 | *egt* | 120151 < 121626 | 148 | 491 (54583) |  | 14 (90) | 16 (76) | 14 (83) | 15 (62) | 138 (80) |
|  | 139 | *lef-1* | 121696 > 122436 | 69 | 246 (28303) |  | 13 (90) | 17 (68) | 13 (74) | 14 (55) | 139 (78) |
|  | 140 |  | 122379 > 123335 | -58 | 318 (36539) |  | 12 (99) | 18 (71) | 12 (75) | 13 (46) | 140 (73) |
|  | 141 |  | 123375 > 124016 | 39 | 213 (23947) |  | 11 (97) | 20 (47) | - | - | - |
|  | 142 |  | 124051 < 125088 | 34 | 345 (40029) |  | 10 (71) | 9 (62) | 11 (71) | 11 (45) | 141 (61) |
|  | 143 | *ptp-1* | 125147 > 125680 | 58 | 177 (20253) |  | 09 (92) | 8 (75) | 10 (81) | 1 (60) | 142 (75) |
|  | 144 | *ptp-2* | 125658 > 126140 | -23 | 160 (18196) |  | 08 (98) | 7 (75) | 9 (73) | - | - |
|  | 145 | *p26a* | 126179 > 126982 | 38 | 267 (30141) |  | 07 (98) | 130 (31) | 132 (32) | 136 (35) | 21 (31) |
|  | 146 |  | 127194 < 127631 | 211 | 145 (16321) |  | 05 (77) | 5 (78) | 8 (82) | 4 (65) | 145 (83) |
|  | 147 |  | 127551 > 127919 | -81 | 122 (13642) |  | 04 (88) | 4 (59) | 7 (79) | 5 (50) | 146 (59) |
|  | 148 | *lef-2* | 127922 > 128539 | 2 | 205 (22813) |  | 03 (86) | 3 (68) | 6 (78) | 6 (55) | 147 (77) |
|  | 149 |  | 128571 > 128954 | 31 | 127 (14749) |  | 02 (83) | 2 (64) | 5 (81) | - | - |
|  |  |  |  |  |  |  |  |  |  |  |  |
